# IDOL in metabolic, neurodegenerative and cardiovascular disease

**DOI:** 10.18632/aging.101597

**Published:** 2018-10-14

**Authors:** Nienke M. van Loon, Patrick C.N. Rensen, Noam Zelcer

**Affiliations:** 1 Department of Medical Biochemistry, Academic Medical Center, University of Amsterdam, 1105AZ Amsterdam, The Netherlands; 2 Department of Medicine, Division of Endocrinology and Einthoven Laboratory for Experimental Vascular and Regenerative Medicine, Leiden University Medical Center, 2333ZA Leiden, The Netherlands

**Keywords:** cholesterol, lipoprotein receptors, E3 ubiquitin ligase, metabolic syndrome, Alzheimer’s disease

Cardiovascular and neurodegenerative diseases, such as atherosclerosis and Alzheimer’s disease (AD), share common risk factors including age and disturbed lipoprotein metabolism. In atherosclerosis, elevated low-density lipoprotein (LDL) cholesterol is a major disease risk factor, while in AD carriers of two ε4 alleles of APOE, the major cholesterol-carrying protein in lipoprotein particles in the central nervous system (CNS), are at a ~12-fold increased risk. Clearance of lipoproteins is a central determinant of their levels and is governed by members of the lipoprotein-receptor family of membrane receptors. Herein, the LDL-receptor (LDLR) plays a prominent role [1], and as such, factors that regulate the level of the LDLR receptor, or of its closely related family members, may influence progression of both cardiovascular and neurodegenerative diseases.

We have previously identified the sterol-regulated Inducible Degrader of the LDL-receptor (IDOL, also known as MYLIP) as a post-transcriptional regulator of the LDLR [2]. Acting as an E3 ubiquitin ligase, IDOL specifically promotes the ubiquitylation and subsequent lysosomal degradation of the LDLR. In addition to the LDLR, IDOL also targets two of its closest family members, the VLDLR and APOER2, which play a prominent role in the Reelin signaling pathway during development of the CNS [3]. The physiological relevance of IDOL in lipoprotein metabolism is supported by genome-wide association studies that have identified variation in the *IDOL* locus to be associated with circulating LDL levels in humans [4]. Consistent with this, we reported that carriers of a loss-of-function variant in *IDOL* have decreased circulating LDL levels, which is in line with enhanced LDL clearance via the LDLR pathway [5]. Similarly, loss of *Idol* in mice, either genetically or through silencing with antisense oligonucleotides (ASO), increases the level of the LDLR in the brain and has been reported to attenuate progression of AD-like symptoms in a mouse model of AD induced by ß-Amyloid aggregation [6]. The phenotypic improvements were proposed to be due to enhanced LDLR-dependent clearance of lipoprotein-associated ß-Amyloid by microglia. While these studies collectively demonstrate the physiological role of IDOL in lipoprotein clearance, its role in whole body energy and lipid metabolism had not been investigated yet

To address this, we recently compared the metabolic phenotype of wildtype and *Idol^(-/-)^* mice during physiological aging and following a metabolic challenge with diet enriched for fat and cholesterol [7]. Remarkably, we found that mice lacking *Idol* are markedly protected from age- and diet-induced alterations commonly associated with the metabolic syndrome. Accordingly, these mice were resistant to developing obesity, dyslipidemia, and hepatic fat accumulation. Maintained brown adipose tissue (BAT) activity may underlie, at least in part, this protection as *Idol^(-/-)^* mice were resistant to accumulation of fat in this tissue. Sustained BAT activity in *Idol^(-/-)^* mice was supported by increased uptake of triglyceride rich lipoprotein-derived fatty acids into this tissue which can fuel thermogenesis. Yet as we suggested [7], our finding of increased locomotion and of increased expression of tyrosine hydroxylase, a proxy for sympathetic activation, in *Idol^(-/-)^* mice and their BAT, respectively, may also suggest involvement of Idol in regulation of energy metabolism in the CNS.

Our study, which was conducted with global *Idol^(-/-)^* mice, raises several intriguing points related to the metabolic role of IDOL. First, this study does not allow us to conclusively pin point the tissue relevant for the protective action of Idol. While we propose that Idol activity in BAT and possibly in the CNS is important for this beneficial outcome, studies with tissue specific ablation of *Idol* will be necessary to establish this conclusively (*e.g.* in BAT). Particularly with respect to the potential role of Idol in the CNS, it would be very important to determine in which cell types and neural circuits it is active and whether its activity is sensitive to metabolic cues. Second, as IDOL can target the LDLR, VLDLR, and APOER2 [3], it will be important to evaluate whether the metabolic effects of IDOL are specifically mediated by one, or a combination of these receptors. Third, in view of IDOL’s role in whole body metabolism and AD, its expression level and activity in tissues in the course of aging and disease progression merits study. To the best of our knowledge this has not been evaluated as yet. Finally, next to ASO-based approaches [6], the recent report of a cyclic peptide that can inhibit IDOL [8] suggests that discovery of small molecules that target IDOL activity is feasible. Given accumulating observations pointing to IDOL as a putative target in both metabolic disease and AD, efforts to develop therapeutic strategies to inhibit its activity are therefore warranted.
